# AgRP/NPY Neuron Excitability Is Modulated by Metabotropic Glutamate Receptor 1 During Fasting

**DOI:** 10.3389/fncel.2018.00276

**Published:** 2018-09-03

**Authors:** Brenton T. Laing, Peixin Li, Cameron A. Schmidt, Wyatt Bunner, Yuan Yuan, Taylor Landry, Amber Prete, Joseph M. McClung, Hu Huang

**Affiliations:** ^1^Human Performance Laboratory, Department of Kinesiology, East Carolina University, Greenville, NC, United States; ^2^East Carolina Diabetes and Obesity Institute, East Carolina University, Greenville, NC, United States; ^3^Department of Physiology, East Carolina University, Greenville, NC, United States

**Keywords:** AgRP/NPY neurons, mGluR1, hypothalamus, fasting, food intake

## Abstract

The potential to control feeding behavior via hypothalamic AgRP/NPY neurons has led to many approaches to modulate their excitability—particularly by glutamatergic input. In the present study using NPY-hrGFP reporter mice, we visualize AgRP/NPY neuronal metabotropic glutamate receptor 1 (mGluR1) expression and test the effect of fasting on mGluR1 function. Using the pharmacological agonist dihydroxyphenylglycine (DHPG), we demonstrate the enhanced capacity of mGluR1 to drive firing of AgRP/NPY neurons after overnight fasting, while antagonist 3-MATIDA reduces firing. Further, under synaptic blockade we demonstrate that DHPG acts directly on AgRP/NPY neurons to create a slow inward current. Using an *in vitro* approach, we show that emulation of intracellular signals associated with fasting by forskolin enhances DHPG induced phosphorylation of extracellularly regulated-signal kinase (1/2) in GT1-7 cell culture. We show *in vivo* that blocking mGluR1 by antagonist 3-MATIDA lowers fasting induced refeeding. In summary, this study identifies a novel layer of regulation on AgRP/NPY neurons integrated with whole body energy balance.

## Introduction

AgRP/NPY neurons are potent regulators of food seeking behavior (Krashes et al., 2011) by release of GABA (Wu et al., 2009), AgRP (Nakajima et al., 2016) or NPY (Paez and Myers, 1991). AgRP/NPY neurons respond to energy deficit (Schwartz et al., 1991; Luquet et al., 2005) due to changes in circulating factors (Chen et al., 2004) and synaptic inputs. Many pre-synaptic sources of glutamate contribute to excitatory activation of AgRP/NPY neurons to drive feeding behavior, such as paraventricular hypothalamic Pituitary Adenylate Cyclase Activating Polypeptide (PACAP) neurons and glutamatergic neurons in the dorsomedial hypothalamus (Krashes et al., 2014). Further, post-synaptic alterations facilitate glutamate receptor driven excitability under the fasted condition (Liu et al., 2012; Qi and Yang, 2015). Synaptic integration occurs in concert with fasting induced changes to intracellular signals of AgRP/NPY neurons, notably including activation of Protein Kinase A (PKA) in arcuate AgRP/NPY neurons (Shimizu-Albergine et al., 2001; Nakajima et al., 2016). In models of metabotropic glutamate receptor 1 (mGluR1), PKA enhances function (Francesconi and Duvoisin, 2000) and promotes surface stability (Mundell et al., 2004). Here, we investigate the interface between intracellular signals of hypothalamic AgRP/NPY neurons and mGluR1 function.

G-protein coupled receptor (GPCR) mGluR1 mRNA is expressed across the adult brain such as hypothalamus, hippocampus, globus pallidus, substantia nigra, thalamus, olfactory bulb, cortex, cerebellum and brain, while mGluR1 protein is expressed mostly in cerebellum, olfactory bulb and hypothalamus (van den Pol, 1994). mGluR1’s are primarily concentrated on post-synaptic structures (Hovelsø et al., 2012) to depolarize the neuron by enhanced inositol triphosphate (IP3) driven release of sequestered intracellular calcium from endoplasmic reticulum. Controversy exists over the presence and function of mGluR1 in the arcuate nucleus of the hypothalamus (van den Pol, 1994; Kiss et al., 1996; Henry et al., 2015; Nestor et al., 2016).

PKA activation facilitates agonist independent signaling of mGluR1 via potentiation of IP3 and raises the ceiling for response to agonist (Francesconi and Duvoisin, 2000). PKA promotes surface stability of mGluR1 by preventing internalization attributable to association with GPCR kinase 2 and arrestin 2 (Mundell et al., 2004). In addition, expression of the mGluR1 scaffold protein Homer 1a (Brakeman et al., 1997) is enhanced by PACAP (Girard et al., 2004). These facilitative effects are notable in light of PKA induced stabilization of α-amino-3-hydroxy-5-methyl-4-isoxazolepropionic acid receptors (AMPARs) by phosphorylation on of the serine 845 (S845) on the GluA1 subunit (Esteban et al., 2003), despite the lack of this residue on mGluR1. The actions of PKA on excitatory drivers stand in stark contrast to PKA-mediated inhibition of G_i_-linked group II and group III metabotropic receptors (Schaffhauser et al., 2000). Taken together, PKA can enhance glutamate receptivity to drive depolarization and blunt inhibitory inputs upon receiving glutamatergic input. This suggests PKA is a prime candidate for the coupling of whole body metabolic status to glutamatergic transmission in AgRP/NPY neurons.

Given reports of fasting induced activation of PKA in arcuate AgRP/NPY neurons (Shimizu-Albergine et al., 2001; Nakajima et al., 2016) paired with reports of no effect by mGluR1 agonist dihydroxyphenylglycine (DHPG) on AgRP/NPY neurons in fed mice (Nestor et al., 2016), a gap in understanding exists regarding if hunger influences mGluR1 function in AgRP/NPY neurons. We hypothesize that mGluR1 modulates excitability of AgRP/NPY neurons specifically under the fasted condition.

We demonstrate that under the fasted condition, mGluR1 plays an excitatory role on AgRP/NPY neurons. Fasting increases AgRP/NPY neuron response to group I metabotropic glutamate receptor agonist DHPG, while we observe no effect of DHPG on AgRP/NPY neurons of fed mice. Similarly, in GT1-7 hypothalamic cells, forskolin stimulation facilitates DHPG induced phosphorylation of ERK1/2. Consistent with this phenomena, enhanced function of mGluR1 occurs as a typical part of fasting as evidenced by a reduction in AgRP/NPY firing rate after 3-MATIDA perfusion onto brain slices and reduced food intake by central administration. Taken together, our data indicate that modulation of neuronal excitability by mGluR1 occurs secondarily to status of the ongoing cell signals.

## Materials and Methods

### Animal Housing and Fasting

B6.FVB-Tg(NPY-hrGFP)1Lowl/J mice were individually housed in accordance with National Institutes of Health Guide for the Care and Use of Laboratory Animals (National Institutes of Health Publication no. 85-23, revised 1996) and approved by the Institutional Animal Care and Use Committees of East Carolina University.

### Fasting Induced Refeeding

Overnight fasting was conducted by placing 3–5 month-old mice in a fresh cage without food while fed animals were moved to a fresh cage and given food *ad libitum*. Alpha-dry bedding was used to prevent consumption of bedding during the fast. mGluR1 inhibitor 3-MATIDA was used for experimentation (Moroni et al., 2002). Food was removed between 5:00 PM and 7:00 PM. On the subsequent day, mice received 1 mg/kg of 3-MATIDA dissolved in DMSO by ICV administration at 7:30 AM (start of light phase) and food was presented at 8:00 AM. Food intake was tracked repeatedly at 30 min intervals for 2 h. Control mice were administered an equivalent amount of DMSO.

### Intracerebroventricular Cannulation

Mice were administered analgesic meloxicam and anesthetized with ketamine and xylazine. Mice were then cannulated for delivery of drug to lateral ventricle by intracerebroventricular cannulation and were included in analysis after post-mortem verification of placement by checking scar tissue from the surface to the lateral ventricle. To implant the cannula, a midline incision enabled visualization of and orientation to the Bregma. A hole was drilled and placed at stereotaxic coordinates (−0.5 mm posterior, 1 mm lateral, 2.5 mm depth) corresponding to mouse lateral ventricle. Two screws were also implanted, approximately at the location of the ipsilateral lambdoid structure and at the contralateral ventricle. 3M carboxylate dental cement was used to secure the implants to the skull. Mice were given at least 2 weeks to recover before conducting experiments.

### Epifluorescence Microscopy and Immunofluorescence

We conducted immunofluorescent imaging as previously described (Laing et al., 2016). Briefly, slices were washed in PBS and blocked in PBS with triton (0.3%). Slices were incubated overnight in primary antibody for cFOS (Santa Cruz; 1:500) or pERK1/2 (Cell Signaling Technology, 1:500). On the subsequent day, slices were washed and incubated in appropriate secondary antibody. After 90 min, slices were washed and mounted for fluorescent microscopy. Co-localization was manually determined by overlaying images and using cell counter plug-in on ImageJ using three closely matched serial sections from three mice in each group.

### Confocal Microscopy

The sections were imaged using an Olympus FV1000 laser scanning confocal microscope (LSCM). Acquisition software was Olympus FluoView FSW (V4.2). The objective used was 60× oil immersion (NA = 1.35, Olympus Plan Apochromat UPLSAPO60X(F)). Images were 800 × 800 pixel with 2 μs/pixel dwell time. Detector noise was reduced by application of a 3× line scanning kalman filter. Images were acquired in sequential scan mode. Dapi was excited using the 405 nm line of a multiline argon laser, emission was filtered using a 490 nm dichroic mirror and 430–470 nm barrier filter. GFP was excited using the 488 nm line of a multiline argon laser, emission was filtered using a 560 nm dichroic mirror and 505–540 nm barrier filter. Alexafluor 594 was excited using a 559 nm laser diode, emission was filtered using a 575–675 nm barrier filter. Standardized laser power and detector gain settings were determined using test sections and zero detector offset was used for all images. Lateral optical resolution was 0.196 μm, and axial optical resolution was 0.902 μm. The pinhole aperture diameter was set to 105 μm (1 Airy disc). A 3× digital zoom was applied to all images used for colocalization analysis to ensure adequate sampling. Lateral pixel size was 0.088 μm/pixel, and axial pixel size was 0.390 μm/slice. Image processing was performed using ImageJ (V1.51f). “Just Another Co-Localization” plug-in was used to identify AgRP/NPY neurons as regions of interest for calculation of Mander’s Overlap Coefficient and Mean Integrated Density. Analysis was conducted on AgRP/NPY neurons from three sections from three mice per group.

### Electrophysiology

We conducted cell attached voltage clamp recordings of AgRP/NPY neurons. Briefly, before 10:00 AM mice were deeply anesthetized by isoflurane followed by intracardial perfusion with chilled N-methyl-D-glucamine solution (in mM: 92 NMDG, 2.5 KCl, 1.25 Nah_2_PO_4_, 30 NaHCO_3_, 20 HEPES, 25 Glucose, 2 Thiourea, 5 Na-Ascorbate, 3 Na-Pyruvate, 0.5 CaCl_2_, 10 MgSO_4_) and sliced into 200–300 μM using VF200 Compresstome (Precisionary Instruments, Greenville NC, USA). Slices recovered for an hour and were stored for recording in a BSK6 (Automate Scientific, Berkley CA, USA) in HEPES solution (in mM: 92 NaCl, 2.5 KCl, 1.25 NaH_2_PO_4_, 30 NaHCO_3_, 20 HEPES, 25 Glucose, 2 Thiourea, 5 Na-ascorbate, 3 Na-pyruvate, 2 CaCl_2_, 2 MgSO_4_). Recordings were conducted in normal aCSF (in mM: 119 NaCl, 2.5 mM KCl, 1.25 mM NaH_2_PO_4_, 24 NaHCO_3_, 12.5 Glucose, 2 CaCl_2_ * H_2_O, 2 MgSO_4_) on a bath (Ting et al., 2014). Neurons were visualized using Leica DM6000FS microscope with polarized differential interference contrast microscopy using infra-red illumination and fluorescent imaging (488 nM). Using 3–7 mOhm pipettes, cell-attached seals were obtained. For cell attached recordings to detect firing rate, gain was modified to enhance signal/noise ratio for clear detection of action potentials and holding potential was set to −50 mV. Seals were stabilized and a 3–5 min baseline period was recorded followed by perfusion of DHPG (50 μM). Firing rates were counted only during recording periods where action potential amplitude exceeded any change in voltage using clampfit threshold counter. Equivalent length periods (0.5–5 min) were set within each recording during perfusion of aCSF or DHPG. Firing rate (Hz) was calculated by dividing the number of events by the number of seconds. For whole cell recordings, gigaohm seals were obtained and the cells were broken into using negative pressure. Current clamp recordings were stabilized for repeated firing under baseline condition. Voltage clamp whole cell recordings were conducted at a −60 mV holding potential.

### Cell Culture

Immortalized hypothalamic GT1-7 cell culture were raised in Dulbecco’s Modified Eagle Medium. GT1-7 cells were pre-treated (1.75 h) with standard media alone, media with adenylyl cyclase stimulant forskolin (10 μM), or media and forskolin (10 μM). Next, we remade pre-treatment mix with or without metabotropic glutamate receptor I (mGlurI) agonist DHPG (50 μM) and applied to cells (0.25 h). Cells were lysed and harvested. Cell Culture Protein concentration from cells within each well was determined using Pierce bicinchoninic acid assay (BCA; Thermo Fisher Scientific). Samples were standardized by adding equal amount of protein and 4× loading buffer combined with variable volume of lysis buffer for western blot.

### Medial Basal Hypothalamic Isolation

Fresh brains were harvested from mice and the medial basal hypothalamus was isolated from the rest of the brain. Briefly, the hypothalamus was removed from the brain with forceps and placed on wet filter paper on an ice-filled tissue culture dish. A blade was used to dissect the ventral portion of hypothalamus by cutting the lower segment of the third ventricle. Finally, lateral portions were dissected and the medial portion was placed in lysis buffer. BCA was conducted to determine protein concentration.

### Western Blot

Equal protein content mixtures were loaded into a 4%–20% HCL gel then transferred to nitrocellulose membrane. Membrane strips were incubated overnight in antibody in 5% milk for pERK (Cell Signaling Technology), total ERK (Cell Signaling Technology), and mGluR1a/b (Santa Cruz). Phosphorylated and Total ERK antibodies were used at a 1:1,000 dilution while mGluR1a/b was used at a 1:50 dilution. Samples were incubated for 2 h in secondary antibody followed by ECL treatment and imaging with Chemidoc. Images were inverted and the mean intensity of from each lane and a blank spot on the strip (background) was measured by using ImageJ. Background was subtracted from mean intensity of each lane. Within each western blot, the measurement box was equivalent size. Phosphorylated ERK was normalized to the mean intensity of total ERK.

### Statistical Analysis

Paired *t*-tests were used to determine differences between firing rate under aCSF and DHPG treatment, while unpaired tests were used for comparisons between fed and fasted neurons as well as for western blots. Statistical significance was determined by *p* < 0.05 (two-tailed). Food intake was analyzed by 2-way repeated measures ANOVA with Sidak correction for multiple comparisons.

## Results

### Hypothalamic AgRP/NPY Neuron mGluR1 Expression

**Figure 1:**
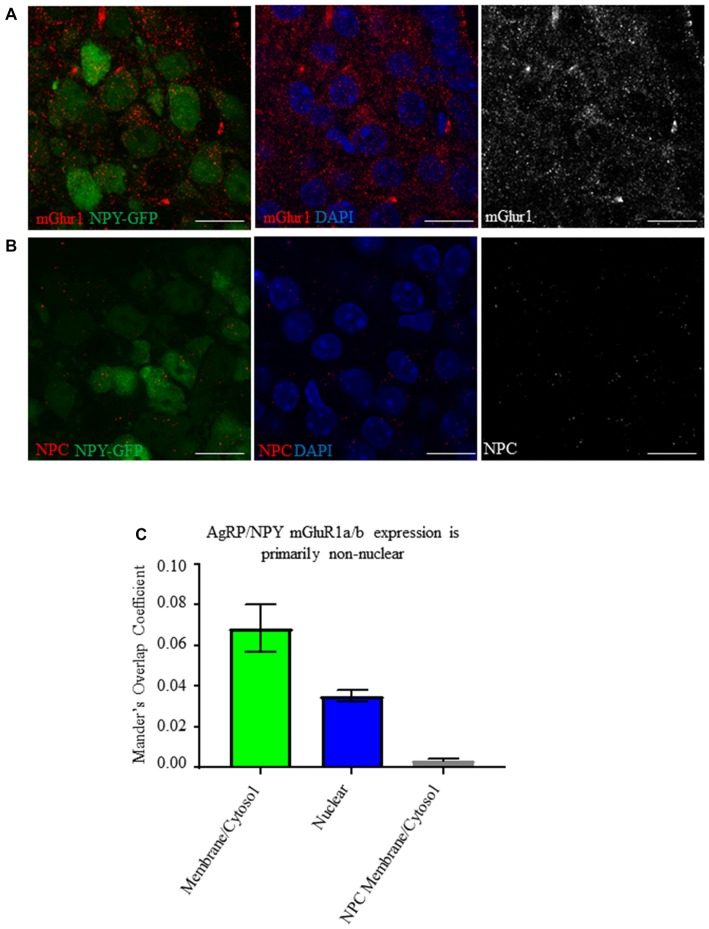
AgRP/NPY neurons express mGluR1a/b. **(A)** Representative images of metabotropic glutamate receptor 1 (mGluR1) a/b (red) expressing AgRP/NPY (green) neurons (*left*), close apposition of mGluR1 (red) to the nucleus of AgRP/NPY neurons (blue; *middle*), and mGluR1 staining alone (gray; *right*). **(B)** Representative images of no primary control stains (red) on AgRP/NPY neurons (green; *left*), nuclei (blue; *middle*), and no primary control gray scale (*right*). **(C)** Manders overlap coefficients for the proportion of cytosol/membrane covered by mGluR1a/b nucleus covered by mGluR1a/b, and cytosol/membrane coverage in the no primary control condition. Scale bars represent 15 μM.

We searched for the presence of mGluR1 on AgRP/NPY neurons using NPY-hrGFP mice and tested for the effect of fasting on intensity of immunoreactivity. Analysis by Mander’s Overlap Coefficient demonstrates mGluR1 immunoreactivity on AgRP/NPY neurons under the fasted condition (Figures 1A,B) that is primarily non-nuclear (Figure 1C). While previous reports show hypothalamic mGluR1a/b (van den Pol, 1994; Mateos et al., 1998), we show visualization of mGluR1a/b specifically on hypothalamic AgRP/NPY neurons.

### Fasting Does Not Alter mGluR1a/b Protein Expression

**Figure 2:**
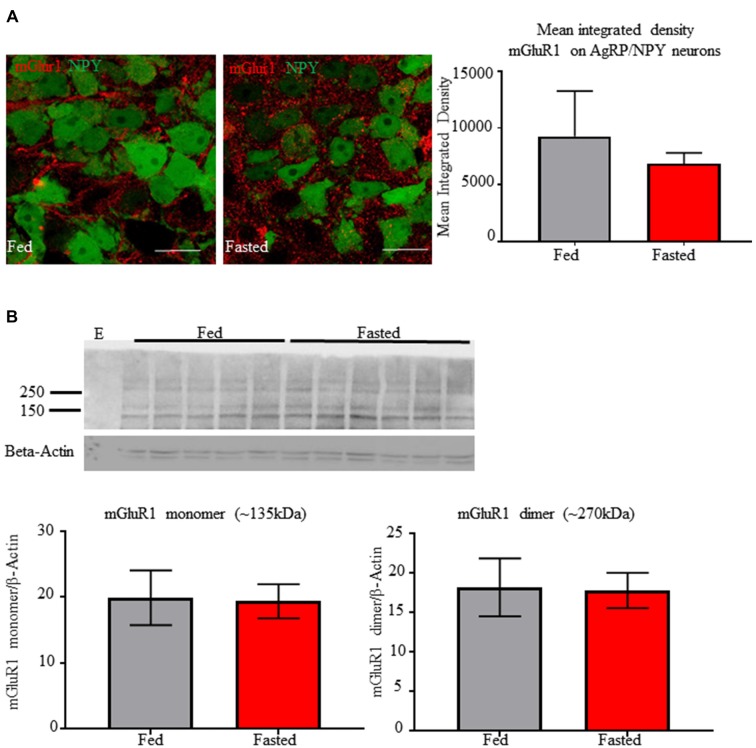
Fasting does not alter immunoreactivity of mGluR1a/b on AgRP/NPY neurons or in detection in homogenate from medial basal hypothalamus. **(A)** Representative confocal images of mGlur1a/b on AgRP/NPY neurons from mice euthanized under fed (left) and fasted (middle) condition, along with quantification (right). **(B)** Western blot of mGlur1a/b and β-Actin from mice euthanized under fed and fasted condition. An empty lane was included on the left as negative control. Quantification of monomer and dimer at approximately 135 kDa and 270 kDa. Scale bars represent 15 μM.

We tested the effect of fasting on mGluR1a/b expression by AgRP/NPY neurons. Co-localization analysis of immunofluorescent images did not reveal any difference in mean integrated density for mGluR1a/b (Figure 2A) between fed (9258 ± 2308) and fasted (6858 ± 548.6) condition. Further, western blot analysis of homogenate taken from medial basal hypothalamus of fed and fasted mice did not reveal any difference in levels of mGlur1a/b monomer (fed = 19.88 ± 4.148, fasted = 19.36 ± 2.574) or dimer (fed = 18.14 ± 3.65, fasted = 17.76 ± 2.23; Figure 2B). Lane 1 was left empty as negative control (E). This is consistent with previous report that food deprivation does not the quantity of mRNA for GRM1 (Henry et al., 2015).

### Fasting Induced AgRP/NPY Activation and Phosphorylation of Extracellular Signal-Regulated Kinase

**Figure 3:**
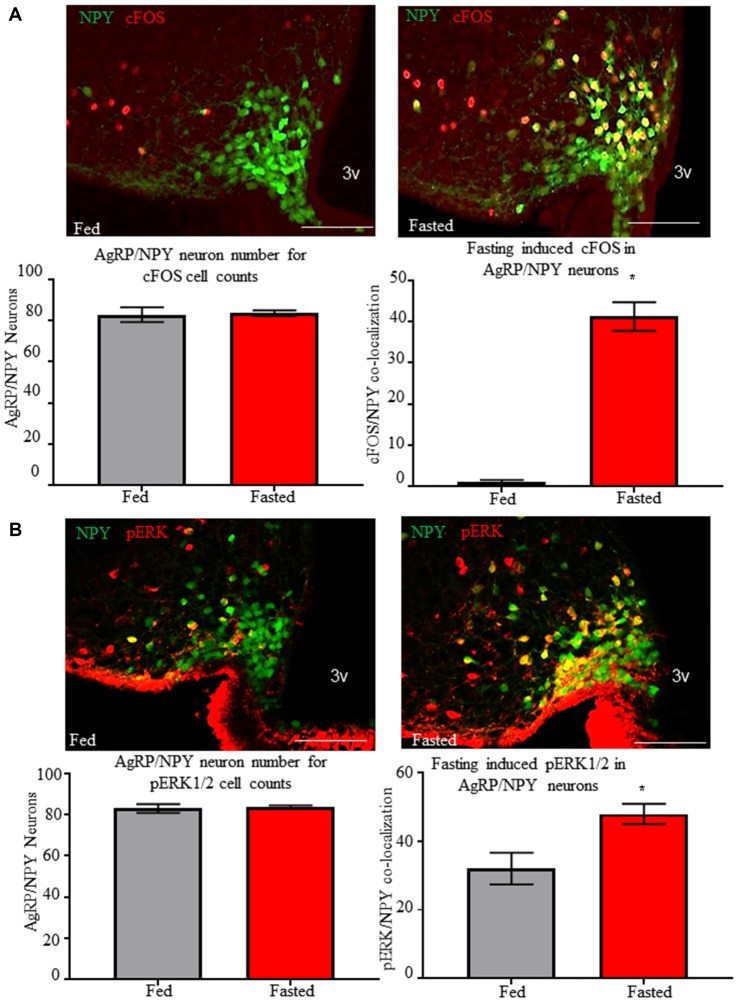
Fasting induces cFOS and phosphorylation of ERK1/2 in AgRP/NPY neurons. **(A)** Representative images of cFOS (red) and NPY/AgRP neurons (green) under fed (*n* = 3) and fasted (*n* = 3) conditions. Count data for mean NPY/AgRP neurons per slice and mean number of AgRP/neurons co-localized with cFOS. **(B)** Representative images of pERK (red) and NPY/AgRP neurons (green) under fed and fasted conditions. Count data for mean NPY/AgRP neurons per slice and mean number of AgRP/NPY neurons co-localized with pERK. Bar graphs show Mean + SEM. *p < 0.05. Scale bars represent 50 μM.

We tested for immunoreactivity of pERK1/2 and cFOS in AgRP/NPY neurons of fed and fasted mice. We observe increased (*p* < 0.05) co-localization of neuronal activation marker cFOS (Figure 3A) from brains of mice euthanized under the fasted (41.22 ± 3.46) compared to the fed (1.11 ± 0.4843) condition. Further, we observe increased phosphorylation of ERK1/2 (Figure 3B) in AgRP/NPY neurons of mice euthanized under fasted (47.89 ± 2.91) compared to fed (32 ± 4.61) condition. This suggests that factors contributing to phosphorylation of ERK1/2, including mGluR1, may be enhanced as part of the activation response to fasting. While previous reports demonstrate that fasting influences arcuate pERK1/2 and NPY immunoreactivity (Morikawa et al., 2004; Ueyama et al., 2004), we show that fasting influences pERK1/2 specifically in AgRP/NPY neurons.

### Forskolin Enhances GT1-7 Cell Responsiveness to Group I mGluR Agonist Dihydroxyphenylglycine

**Figure 4:**
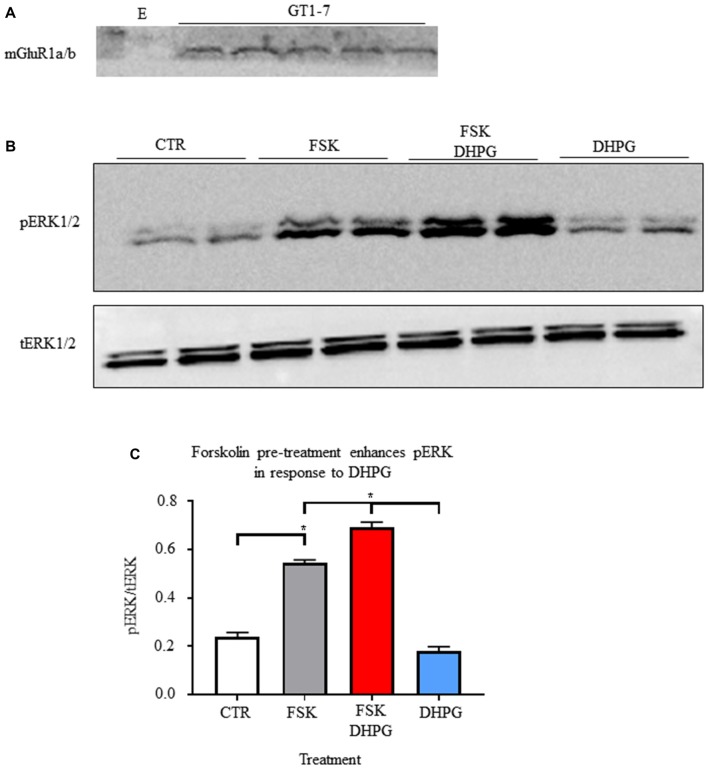
Forskolin enhances group I metabotropic receptor function. **(A)** Representative western blot of mGlur1a/b detection in samples from GT1-7 cells. **(B)** Representative images of western blots for phosphorylation of ERK (1/2) and total ERK (1/2). **(C)** Bar graph of mean intensity for phosphorylated ERK (1/2; *n* = 4) normalized to total ERK (*n* = 3). Student’s *t*-test used for analysis of pre-determined comparisons between conditions. *p < 0.05.

We used adenylyl cyclase stimulant forskolin to mimic fasting induced AgRP/NPY neuron intracellular signals and their action on mGluR1a/b. As previously reported (Sortino et al., 1996), we confirmed that GT1-7 cells express mGluR1a/b (Figure 4A). Given the utility of ERK1/2 activity as a readout for Gq Protein-Coupled Receptor manipulation (Osmond et al., 2005), we tested for the effect of mGluR1 agonism by DHPG (0.6892 ± 0.0223) with and without forskolin pre-treatment (0.1816 ± 0.01752) compared to control (0.2388 ± 0.0186) and forskolin alone (0.5442 ± 0.0122). We show *in vitro* that pre-treatment with forskolin enhances mGluR1 function by DHPG-induced phosphorylation of ERK1/2 (*p* < 0.05; Figures 4B,C). Of note, treatment with DHPG alone did not significantly increase pERK1/2. This data demonstrates that function of group I mGluRs occurs in accordance with ongoing intracellular signals.

### Fasting Enhances AgRP/NPY Responsiveness to Group I mGluR Agonist Dihydroxyphenylglycine *ex vivo*

**Figure 5:**
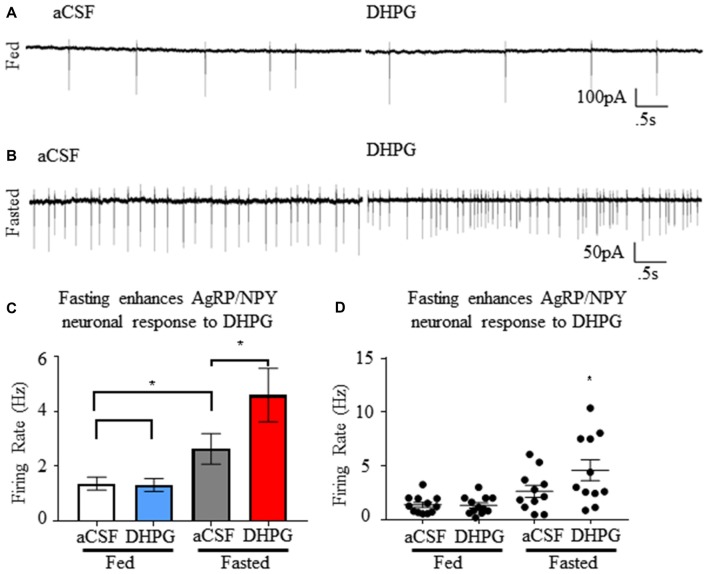
Group I metabotropic receptor agonist dihydroxyphenylglycine (DHPG) enhances firing rate of NPY/AgRP neurons specifically under the fasted condition. **(A)** Representative trace showing neuronal firing of AgRP/NPY neuron from a fed mouse under aCSF and DHPG. **(B)** Representative trace showing neuronal firing of AgRP/NPY neuron from fasted mouse under aCSF (middle left) and DHPG (middle right). **(C)** Bar graph (bottom left) of mean firing rate and **(D)** dot plot of each individual neuron (bottom right). Standard *t*-test used to compare across fed and fasted conditions, matched pairs *t*-test used for detection of within condition differences. Means ± SEM (*n* = 11 fed; *n* = 12 fasted). Bar graph significance marked by *p < 0.05.

Using cell attached recordings of AgRP/NPY neurons, we confirmed that mGluR1 agonist DHPG (50 μM) has no effect on firing rate of AgRP/NPY neurons from mice under the fed status (*n* = 12; Figure 5A), but that DHPG enhanced firing rate of AgRP/NPY neurons from mice under the fasted condition (*n* = 11; Figure 5B). Our data showing no observable effect of DHPG on AgRP/NPY neurons from fed mice is consistent with a previous report that there is no change in AgRP/NPY membrane potential by DHPG (Nestor et al., 2016). Notably, mGluR1 function after fasting can enhance neuronal firing of AgRP/NPY neurons even beyond the typically high fasting-induced firing rate (Figures 5C,D). Because this pharmacological stimulation well exceeds normal glutamatergic inputs available to mGluR1, this strongly suggests that mGluR1 has the capacity to drive excitability under appropriate conditions.

### Group I Metabotropic Receptor Activation Induces a Slow Inward Current in Synaptically Isolated AgRP/NPY Neurons

**Figure 6:**
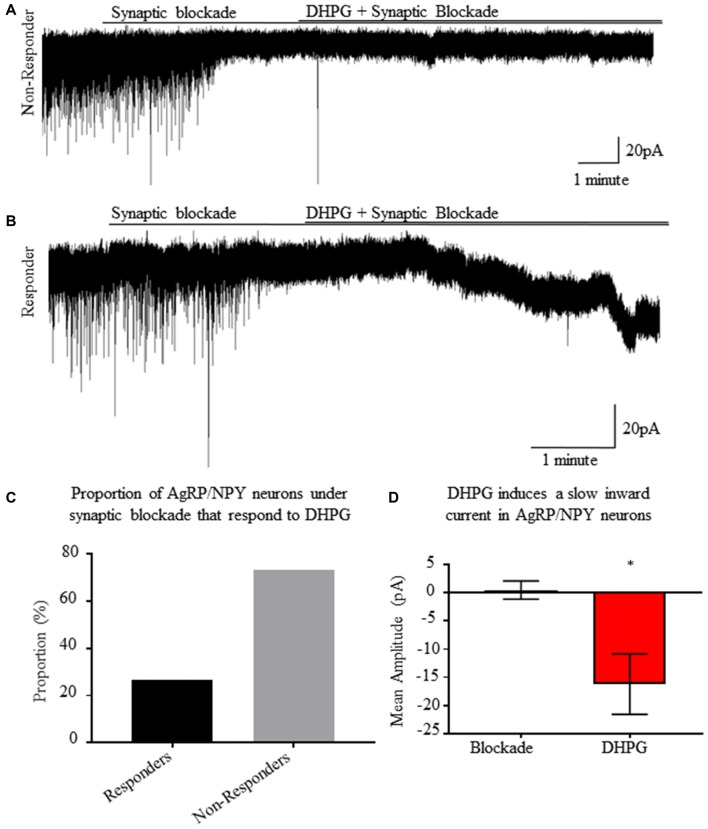
A subset of AgRP/NPY neurons under synaptic blockade exhibit a slow inward current in response to group I metabotropic receptor agonist DHPG. **(A)** Representative whole cell voltage clamp recording of an AgRP/NPY neuron that does not respond to DHPG. **(B)** Representative whole cell voltage clamp recording of an AgRP/NPY neuron in response to DHPG. **(C)** Bar graph of proportion of AgRP/NPY neurons from fasted mice that are DHPG responders (4/16). **(D)** Bar graph of current change from previous condition. Matched pair *t*-test to compare blockade and blockade + DHPG conditions. Means ± SEM (*n* = 4). Bar graph significance marked by *p < 0.05.

Using whole cell voltage clamp recordings from AgRP/NPY neurons of fasted mice, we employed an ionotropic synaptic blockade with AP5, CNQX and picrotoxin prior to perfusion of DHPG (Figure 6A). Once isolated, DHPG perfusion results in slow excitatory currents (Figure 6B) in a subset of AgRP/NPY neurons (*n* = 4/16; Figure 6C). The slow current observed occurs over the timescale of minutes as previously described under previous measurements of mGluR1 induced slow current (Heinbockel et al., 2004; Dong and Ennis, 2018). The average amplitude of observed slow currents is greater than 10 pA (Figure 6D), which is a physiologically relevant influence on small neurons such as AgRP/NPY neurons (Baver et al., 2014).

### mGluR1 Antagonism Reveals Contribution to AgRP/NPY Neuron Activation During Fasting

**Figure 7:**
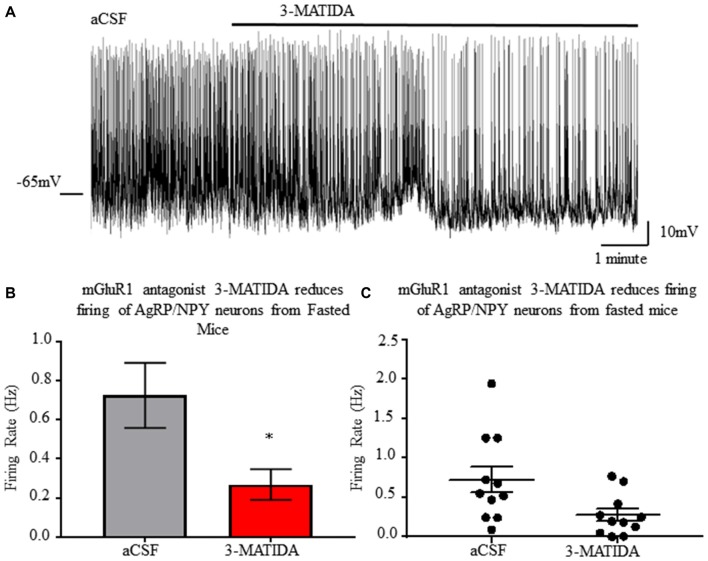
mGluR1 antagonist 3-MATIDA slows firing rate of NPY/AgRP neurons from fasted mice. **(A)** Representative whole cell recordings of neuronal firing of AgRP/NPY neuron from a fed mouse under aCSF and 3-MATIDA. **(B)** Bar graph of mean firing rate. **(C)** Dot plot of each individual neuron. Matched pair *t*-test used to compare across fed and fasted conditions. Means ± SEM (*n* = 11). Bar graph significance marked by *p < 0.05.

Bath application of mGluR1 antagonist 3-MATIDA (100 μM) reduces AgRP/NPY firing rate (*p* < 0.05; Figure 7) from brain slices (*n* = 11) of fasted mice. Because loss of mGluR1 function results in slowed firing, this data indicates mGluR1 contributes to increased fasting induced firing rate. This may co-occur with ionotropic glutamatergic function as reported elsewhere (Liu et al., 2012).

### Central mGluR1 Antagonism Reduces Fasting Induced Refeeding

**Figure 8:**
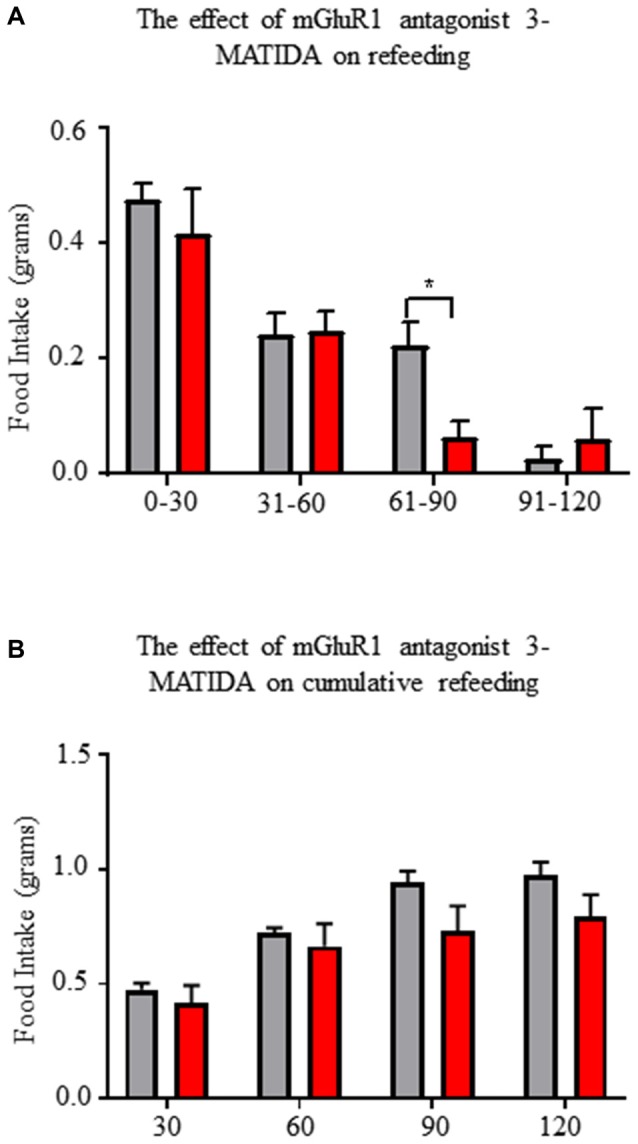
Central administration of mGluR1 antagonist 3-MATIDA reduces fasting induced refeeding. **(A)** Food intake values over 2 h of refeeding broken into 30 min epochs. **(B)** Cumulative food intake values over 2 h. Repeated measure two-way ANOVA with Sidak multiple comparison test was used. *p < 0.05, *n* = 5 for each group.

Next, we tested for a role of mGluR1 on AgRP/NPY neurons to control behavior. Based on the excitatory action of mGluR1 and the established role of AgRP/NPY neurons to control food seeking behavior during fasting, we tested the effect of mGluR1 antagonism during a fasting induced refeeding assay. Central administration of mGluR1 antagonist 3-MATIDA reduces food intake at the 60–90 min mark during fasting induced refeeding (Figure 8A), but there is no change in cumulative refeeding (Figure 8B). Given that AgRP/NPY neurons are essential for ghrelin to drive feeding behavior (Chen et al., 2004), it is notable that this time coincides with reported decreases in ghrelin levels (Gomez et al., 2004).

## Discussion

In the present study, we demonstrate that overnight fasting enhances function of mGluR1 on AgRP/NPY neurons in the hypothalamus, and that central antagonism of this receptor reduces refeeding behavior. Many previous reports have investigated the influence of hormonal and synaptic changes that occur in response to fasting. For the first time, we relate fasting induced intracellular signals of AgRP/NPY neurons to enhance post-synaptic mGluR1 function.

With multiple reports of mGluR1detection in medial basal hypothalamus (van den Pol, 1994; Mateos et al., 1998), to our knowledge, this report is the first to visualize mGluR1 localization specifically on AgRP/NPY neurons. Expression of mGluR1 is consistent with the presence of mRNA for GRM1 (Henry et al., 2015). Consistent with this finding, our imaging and western blot data indicate that AgRP/NPY expression of mGluR1 does not change in the fasted status. While other cells in the arcuate nucleus also express mGluR1 (Nestor et al., 2016), the potent role of AgRP/NPY neurons to control energy balance makes them a key target for investigation of mGluR1 function. At this time, it is unclear how mGluR1 function becomes enhanced under the fasted status without any change in levels of expression. While many factors contribute to the activation of AgRP/NPY neurons during fasting, our observation of coupling between increased cFOS and phosphorylated ERK indirectly point to a contribution by mGluR1 under fasted status.

AgRP/NPY neurons exhibit responsiveness to mGluR1 agonist DHPG in AgRP/NPY neurons from mice euthanized under the fasted but not fed condition. This is consistent with previous reports that DHPG agonism has no effect on AgRP/NPY neurons of fed mice (Nestor et al., 2016). This finding demonstrates that fasting enhances the capacity of mGluR1to mediate AgRP/NPY neuronal firing under physiological conditions in response to pharmacological stimulation. A change in function highlights the possibility that fasting results in trafficking to favorable subcellular locations, improved surface stability, or improved coupling to Gq-GPCR machinery.

Consistent with visual evidence for localization of mGluR1 on AgRP/NPY neurons and the observed change in firing rate, we report functional post-synaptic effects of DHPG directly on AgRP/NPY neurons. Voltage clamp electrophysiology revealed a DHPG-induced slow excitatory post-synaptic current in a subset of AgRP/NPY neurons of fasted mice under ionotropic synaptic blockade, similar to the slow-current observed in cells in the olfactory bulb (Heinbockel et al., 2004; Dong and Ennis, 2018). Given reports that a 10 pA change to the rheobase of small neurons like AgRP/NPY neurons can result in substantial changes to their level of activation (Baver et al., 2014), our observed change demonstrates a physiologically relevant slow current which can influence excitability of AgRP/NPY neurons. This indicates that fasting enhances the effects of glutamatergic inputs to drive activation of AgRP/NPY neurons.

PKA is a critical regulator of leptin action in the hypothalamus (Yang and McKnight, 2015) and is critical for the calcium response to ghrelin (Kohno et al., 2003) whereby blockade of PKA activity with H89 reduces the effectiveness of AgRP/NPY neurons to repeatedly respond to ghrelin. A number of groups have reported PKA activation in AgRP/NPY neurons of mouse hypothalamus during fasting (Shimizu-Albergine et al., 2001; Morikawa et al., 2004; Ueyama et al., 2004), and that forskolin stimulates AgRP transcription in GT1-7 cells via PKA activation (Nakajima et al., 2016). Notably, in HEK293 cells transfected to express mGluR1, forskolin enhances mGluR1 response to glutamate (Francesconi and Duvoisin, 2000), likely by preventing internalization of mGluR1 by reducing association with GPCR kinase 2 and arrestin 2 (Mundell et al., 2004) and may facilitate surface expression by direct action by PKA to mask RRKK domain on the C-terminus of mGluR1 (Tateyama and Kubo, 2008). Loss of GPCR kinase 2 has similar excitotoxic effects as overactive mGluR1, further suggesting that this may be a key regulatory point by PKA (Degos et al., 2013). Thus, we used forskolin to mimic the fasting induced intracellular signals and this resulted in enhanced mGluR1 induced p-ERK. This effect isn’t explained by simple addition of forskolin or DHPG treatment. This *in vitro* experiment is consistent with enhanced function of mGluR1 in hypothalamic neurons in concordance with increased PKA activity during the fasted state.

In AgRP/NPY neurons of mice euthanized under the fasted condition, *ex vivo* experiments with antagonist 3-MATIDA reveal a reduction in neuronal firing rate compared to aCSF perfusion. Slowed firing in response to 3-MATIDA establishes a physiological role of mGluR1 for control of AgRP/NPY neurons. A reduction of firing by AgRP/NPY neurons indicated that 3-MATIDA may have a role in reducing feeding.

mGluR1 antagonism with central administration of 3-MATIDA blunts re-feeding behavior at a 60–90 min time point. Within the first 60 min of re-feeding after an overnight fast, ghrelin is the dominant driver of hunger, but previous reports demonstrate that by 60 min circulating ghrelin levels have waned (Gomez et al., 2004). Decreased availability of ghrelin results in a smaller pool of activated AgRP/NPY neurons. Once the pool of AgRP/NPY neurons falls below a critical point of around 800 neurons (Aponte et al., 2011), food seeking behavior becomes reduced. Our data suggests that mGluR1 serves as a modulator of AgRP/NPY excitability, likely by maintaining the pool of activated AgRP/NPY neurons above threshold required to drive feeding. Loss of this excitatory drive by antagonism of mGluR1 results in reduced hunger that becomes apparent as measured by a reduction in feeding behavior.

Some limitations arose throughout the course of this study. First, food intake experiments were conducted using 3-MATIDA at a dosage that may also interfere with other glutamate receptor signaling. Future studies should aim to test the effects of cutting edge negative allosteric modulators which have superior specificity. Likewise, positive allosteric modulators could be used in place of DHPG to verify that the agonist effects are specific to mGluR1 and not mGluR5, and the role of mGluR1 in AgRP neuron to regulate feeding behavior under fasting vs. fed condition also needs to be addressed in the future by generating AgRP neuron specific mGluR1 deficient mouse model. Second, estimating the proportion of AgRP/NPY neurons that express mGluR1 was unachievable by immunofluorescent methods. Future studies could use single-cell RT-PCR on a number of AgRP/NPY neurons to determine how many could potentially express the receptor, but this also is inadequate to measure functional mGluR1 available at the synapse. In addition, use of single-cell RT-PCR to determine the molecular identity of agonist responders and non-responders may provide further insight into why only a subset respond with slow current.

Approaches targeted at altering function of mGluR1 modulation may hold the potential to be a new frontier for realization of benefits from the potential of existing treatments. Disorder of excitatory synaptic transmission via metabotropic glutamate receptors contributes to nervous system diseases ranging from chronic pain, Cancer, Autism, Schizophrenia, Alzheimer’s disease, Parkinson’s disease, neuroinflammation, Multiple Sclerosis, Epilepsy, Stroke, Obesity and Diabetes—the diversity of these diseases owed to the broad regional distribution of metabotropic glutamate receptors. Clinical trials to test employment drugs to manipulate mGluR1 have yielded limited results for treatment of epilepsy, pain, Alzheimer’s, Parkinson’s, anxiety/depression (Hovelsø et al., 2012).

In summary, our results demonstrate a functional role of mGluR1 on AgRP/NPY neurons and that mGluR1 contributes to feeding behavior. Our results demonstrate the underpinnings for a novel approach to hunger control via modulation of AgRP/NPY neuron excitability by control of intracellular signals responsible for mGluR1 activation.

## Author Contributions

HH and BL designed the study. BL performed most of electrophysiology recording experiments with NPY-GFP reporter mice. PL and TL helped cell culture and western blot experiment. AP helped on cells counting. CS and JM helped on 2-photon microscopy. WB and YY bred and maintained experimental mice.

## Conflict of Interest Statement

The authors declare that the research was conducted in the absence of any commercial or financial relationships that could be construed as a potential conflict of interest.
